# Increased Plasma Level of 24S-Hydroxycholesterol and Polymorphism of CYP46A1 SNP (rs754203) Are Associated With Mild Cognitive Impairment in Patients With Type 2 Diabetes

**DOI:** 10.3389/fnagi.2021.619916

**Published:** 2021-05-13

**Authors:** Jijing Shi, Jianhong Jia, Sai Tian, Haoqiang Zhang, Ke An, Wenwen Zhu, Wuyou Cao, Yang Yuan, Shaohua Wang

**Affiliations:** ^1^Department of Endocrinology, Affiliated Zhongda Hospital of Southeast University, Nanjing, China; ^2^School of Medicine, Southeast University, Nanjing, China; ^3^Department of Endocrinology, Siyang Hospital of Traditional Chinese Medicine, Suqian, China

**Keywords:** type 2 diabetes mellitus, executive function, attention function, mild cognitive impairment, 24S-hydroxycholesterol

## Abstract

**Background:**

Abnormal cholesterol metabolism is common in type 2 diabetes mellitus (T2DM) and causes dementia. Cholesterol 24S-hydroxylase (CYP46A1) converts cholesterol into 24S-hydroxycholesterol (24-OHC) and maintains cholesterol homeostasis in the brain.

**Objective:**

This study aimed to investigate the roles of 24-OHC and the CYP46A1 (rs754203) polymorphism in patients with T2DM and mild cognitive impairment (MCI).

**Methods:**

A total of 193 Chinese patients with T2DM were recruited into two groups according to the Montreal Cognitive Assessment (MoCA). Demographic and clinical data were collected, and neuropsychological tests were conducted. Enzyme-linked immunosorbent assay (ELISA) and Seqnome method were used to detect the concentration of plasma 24-OHC and the CYP46A1 rs754203 genotype, respectively.

**Results:**

Compared with 118 healthy cognition participants, patients with MCI (*n* = 75) displayed a higher plasma level of 24-OHC and total cholesterol concentration (all *p* = 0.031), while no correlation was found between them. In the overall diabetes population, the plasma level of 24-OHC was negatively correlated with MoCA (*r* = −0.150, *p* = 0.039), and it was further proved to be an independent risk factor of diabetic MCI (OR = 1.848, *p* = 0.001). Additionally, patients with MCI and the CC genotype of CYP46A1 rs754203 showed the highest plasma level of 24-OHC even though the difference was not statistically significant, and they obtained low scores in both the verbal fluency test and Stroop color and word test A (*p* = 0.008 and *p* = 0.029, respectively).

**Conclusion:**

In patients with T2DM, high plasma level of 24-OHC and the CC genotype carrier of CYP46A1 rs754203 may portend a high risk of developing early cognitive impairment, including attention and executive deficits.

## Introduction

Type 2 diabetes mellitus (T2DM) has become one of the global public health events with rapidly increasing incidence, and its chronic complications, including cognitive impairment, seriously endanger the patients. As a cognitive disorder syndrome between normal aging and dementia (Korolev et al., 2016; Kaneko et al., 2019), mild cognitive impairment (MCI) is the pre-clinical state of Alzheimer’s disease (AD). Given that adults with MCI tend to develop AD more rapidly than people of the same age who have no cognitive impairment (Petersen et al., 2001), approximately 10–15% of adults with MCI progress to AD per year (Petersen et al., 1999, 2001; Mitchell and Shiri-Feshki, 2009). Moreover, strong epidemiological studies have shown that diabetes is closely related to the occurrence of cognitive impairment (Gudala et al., 2013; Koekkoek et al., 2015; Zhang et al., 2017), and the incidence of MCI is higher in individuals with T2DM than in those without diabetes (Yuan and Wang, 2017). Thus, early diagnosis of MCI is crucial to prevent AD, especially in the high-risk diabetic population. To date, the mechanism of diabetes-associated MCI has not been fully elucidated. Many factors may be involved, such as high oxidative stress, mitochondrial dysfunction, dyslipidemia, insulin resistance, and impaired insulin signaling (Ahmad, 2013; Jayaraj et al., 2020).

Patients with diabetes are often accompanied with changes in cholesterol metabolism (Monnier et al., 1995). Previous research reported that insulin and diabetes can alter cholesterol metabolism in the brain (Suzuki et al., 2010). The brain is rich in cholesterol, accounting for 25% of the total body cholesterol (Björkhem and Meaney, 2004). Meanwhile, cholesterol metabolism was found to be related to the production and accumulation of aβ and the phosphorylation of tau protein (Yanagisawa, 2002), both of which play important roles in the occurrence of cognitive impairment. On the basis of the above-mentioned points, diabetes, cholesterol metabolism, and MCI are closely connected to one another. Cholesterol in the brain is produced *in situ* (Kacher et al., 2019). Cholesterol does not cross through the blood–brain barrier, and the brain’s cholesterol level is unaffected by peripheral cholesterol levels (Chobanian and Hollander, 1962; Jin et al., 2019). Therefore, special methods are needed to remove excess cholesterol from the brain so as to maintain the balance of cholesterol in the brain. Multiple enzymatic reactions can be considered to eliminate cholesterol in the brain, of which cholesterol 24-hydroxylation by CYP46A1 is quantitatively significant, accounting for the elimination of 75–85% of excess cholesterol from humans (Björkhem et al., 1998; Petrov and Pikuleva, 2019). Through cholesterol 24S-hydroxylase encoded by the CYP46A1 gene, cholesterol in the brain is converted into 24S-hydroxycholesterol (24-OHC), which is neurotoxic and can induce nerve cell death (Yamanaka et al., 2011). More than 90% of 24-OHC enters the blood through the blood–brain barrier (Kölsch et al., 2009). Cholesterol synthesis in the brain is balanced by the excretion of 24-OHC (Lund et al., 2003; Björkhem and Meaney, 2004). Meanwhile, more than 90% of 24-OHC in circulation is generated in the brain and can reflect the dynamic balance of cholesterol metabolism in the brain (Papassotiropoulos et al., 2000; Björkhem, 2006).

As a member of the human cytochrome P450 family, CYP46A1 plays an important role in the pathogenesis of both diabetes and cognitive decline. The polymorphism of CYP46A1 rs754203 is closely related to the risk of T2DM onset (Rizvi et al., 2017), which also participates in the risk of AD incidence (Li et al., 2013). However, the relationship between the polymorphism of CYP46A1 rs754203 and AD remains controversial, with some studies confirming the link and others disproving it (Chalmers et al., 2004; Jin et al., 2013). Interestingly, patients with AD and the CYP46A1 TT genotype seem to have the highest level of plasma 24-OHC (Li et al., 2018), which suggests that the CYP46A1 rs754203 polymorphism may play a role in mediating the increased risk of AD by causing differences in blood 24-OHC concentration levels.

Although numerous studies have proved that CYP46A1 and its rs754203 polymorphisms play an etiologic role in T2DM and MCI, their involvement in diabetic MCI has not yet been elucidated. By comparing the plasma level of the 24-OHC concentration and the CYP46A1 rs754203 polymorphism in patients with T2DM with or without MCI, we aimed to clarify whether there is a significant difference in the plasma level of the 24-OHC concentration between the two groups and what role the CYP46A1 rs754203 polymorphism plays in the occurrence of MCI. The results of this work can help reveal how abnormal cholesterol metabolism participates in the occurrence of MCI in T2DM and identify the early warning signs of diabetic MCI.

## Materials and Methods

### Study Population and Study Design

The study was conducted among 193 patients with T2DM who were hospitalized in the Department of Endocrinology, Zhongda Hospital, Southeast University. All participants were of Chinese Han nationality and signed an informed consent form based on a program approved by the Research Ethics Committee of the Affiliated Zhongda Hospital of Southeast University. All methods were carried out in accordance with relevant guidelines and regulations.

We recruited a total of 193 patients diagnosed with T2DM based on the 1999 World Health Organization criteria for diabetes, the duration of which is greater than or equal to 3 years (Alberti and Zimmet, 1998). The patients were 40–75 years old and fully cooperated to finish the test in this study. According to the Montreal Cognitive Assessment (MoCA), a brief and sensitive screening test for MCI (Dautzenberg et al., 2020), we divided the participants into two groups: 75 were patients with diabetes and MCI (MoCA < 26) and 118 were patients with diabetes and normal cognitive function (MoCA ≥ 26). All of the patients with MCI satisfied the diagnostic criteria proposed by the MCI Working Group of the European Consortium on AD in 2006 (Portet et al., 2006) as follows: (1) cognitive complaints from patients or their families, (2) decline in cognitive function in the past year relative to previous abilities, as reported by the patient or an informant, with a clinical dementia rating score of 0.5, (3) cognitive disorders as evidenced by clinical evaluation (impairment in memory or in another cognitive domain), (4) the absence of major repercussions in daily life (however, a patient may report difficulties in performing complex day-to-day activities), and (5) the absence of dementia. The following exclusion criteria were considered in this study: acute complications of diabetes such as hypoglycemia coma, diabetic ketoacidosis, lactic acidosis, and hyperosmolar non-kenotic diabetic coma; cerebrovascular accident confirmed by cranial imaging scan in the past year; definitive diagnosis of neurodegenerative diseases, such as AD and Parkinson’s disease; suffering from depression within 2 months; history of drug abuse or dependence; use of psychotropic drugs within 3 months prior to screening (e.g., anti-Parkinson’s drugs, benzodiazepines, sedatives, and anti-epilepsy drugs); systemic diseases such as malignant tumors, anemia, and severe infections; thyroid diseases; and visual and auditory defects that prevent neuropsychological testing.

### Clinical Data Collection

Demographic variables including age, sex, education, smoking, drinking history, height, weight, and contact information were obtained through standardized interviews. Detailed medical information such as the diabetes duration, use of insulin, and hypertension (including the duration of hypertension) was recorded. Data on weight and height were collected to calculate the body mass index: BMI = weight/height^2^ (kg/m^2^). Patients with systolic blood pressure ≥ 140 mmHg or diastolic blood pressure ≥ 90 mmHg are defined as hypertensive. Fasting blood samples were collected to measure fasting blood glucose (FBG), glycosylated hemoglobin (HbA1c,%), total cholesterol (TC), triglycerides (TG), low-density lipoprotein cholesterol (LDL-C), high-density lipoprotein cholesterol (HDL-C), apolipoprotein A1 (ApoA-1), and apolipoprotein B (ApoB). Blood glucose at 2 h after a meal (2h-PBG) was also measured. All the experimental data mentioned above were obtained from the laboratory of the Affiliated Zhongda Hospital of Southeast University.

### Neuropsychological Tests

The participants’ cognitive function including attention, immediate memory, delayed memory, semantic memory, executive function, psychomotor speed, and visual–spatial ability was assessed *via* some neuropsychological tests. MoCA was first carried out to determine whether the participant is defined as MCI; the participant who had less than 12 years of education was added an extra point to obtain the adjusted MoCA score (Nasreddine et al., 2005). Other neuropsychological tests composed of the mini-mental state exam (MMSE) (Folstein et al., 1975), digit span test (DST) (Leung et al., 2011), verbal fluency test (VFT) (Teng et al., 2013), clock drawing test (CDT) (Shulman, 2000), auditory verbal learning test (AVLT) (Can et al., 2016), logical memory test (LMT) (Chapman et al., 2016), Stroop color and word test (SCWT) (Scarpina and Tagini, 2017), and trail making tests A and B (TMT-A and TMT-B) (St-Hilaire et al., 2018) were also conducted. Each participant’s test lasted about 45 min. All the tests were conducted by an experienced neuropsychologist from the Zhongda Hospital of Southeast University. The neuropsychologist and all subjects were blinded to the study design.

### Measurement of Plasma 24-OHC

Blood samples were obtained from the participants after overnight fasting and stored in tubes with EDTA. The plasma was then stored at −80°C until assay. The plasma 24-OHC concentration of all specimens was detected by Enzyme-linked immunosorbent assay (ELISA) kits on the same day (Nanjing Jin Yibai Biological Technology Co., Ltd.) according to the instructions of the manufacturer. The final dilution of the sample was fivefold. The detection range of the kit used was 1.2–42 ng/L. The intra- and inter-assay coefficients of variation were less than 9 and 11%, respectively.

### Genotyping of the CYP46A1 rs754203 Polymorphism

CYP46A1 SNP (rs754203) was detected by the Sequenom method (CNKINGBIO, Beijing, China). First, DNA was extracted from blood samples by using a DNA purification kit (Puregene, Gentra System, Minneapolis, MN, United States) following the manufacturer’s instructions. Second, the target regions were amplified by PCR, and the PCR products were processed with iPLEX^®^ Pro or iPLEX Gold reagents. Third, a manual pipettor was used to transfer the samples from microtiter plates to a SpectroCHIP^®^Array. Data that had been saved to the MassARRAY database were obtained from the SpectroCHIP^®^ array by using the MassARRAY^®^ Analyzer. Finally, with the help of Typer software, the data were automatically analyzed, and a genotyping report was acquired.

### Statistical Analysis

Data were expressed as mean ± SD, median (interquartile range), or percentage. Student’s *t*-test and ANOVA were used for the analysis of normally distributed variables, with non-parametric Mann-Whitney *U* and Kruskal-Wallis tests used to analyze asymmetrically distributed variables. Chi-square (χ^2^) test was used to compare qualitative variables, explore the distribution of genotypes and allele frequencies, and determine deviations from the Hardy–Weinberg equilibrium. To examine the correlation between neuropsychological test scores and plasma 24-OHC concentrations, we used partial correlation. Logistic regression was conducted to identify independent variables that can significantly increase the risk of having MCI in patients with T2DM. SPSS 22.0 was conducted for statistical analysis (IBM Corporation). A *p*-value < 0.05 indicated statistical significance.

## Results

### Demographic, Clinical, and Cognitive Characteristics

The baseline information of the patients with T2DM recruited in the research is shown in Table 1. Of the 193 patients enrolled in the study, 75 had MCI and 118 were cognitively normal. Patients with MCI were older, had a shorter education time, and suffered longer with hypertension than those with normal cognition. There was a difference in gender distribution between the two groups: more males were in the normal group, while more females were in the MCI group. Compared with the normal cognition group, the MCI group displayed higher levels of HbA1c and TC. A significant difference in the plasma 24-OHC concentration was found between the two groups (*p* = 0.031). Besides the characteristics mentioned above, other indicators such as history of smoking and drinking, BMI, percentage of patients with hypertension, SBP, DBP, diabetes duration, FBG, 2h-PBG, TG, LDL-c, HDL-c, use of insulin, ApoA1, and ApoB all showed no significant difference between the two groups (*p* > 0.05). In the neuropsychological test scores, except SCWT A number, significant differences were observed between the two groups, with high scores in the control group and low scores in the MCI group (*p* < 0.05).

**TABLE 1:** Demographic, clinical, and cognitive characteristics of patients.

	MCI (*n* = 75)	Normal (*n* = 118)	*p*-Value
Age (years)	61.52 ± 8.43	58.43 ± 8.92	0.018a*
Gender (male/female)	32/43	79/39	0.001c*
Education (years)	9.39 ± 3.61	10.83 ± 3.04	0.003a*
Ever smoked, *n* (%)	20 (26.70)	42 (35.60)	0.195c
Drinking, *n* (%)	12 (16.00)	25 (21.20)	0.372c
BMI (kg/m^2^)	24.91 ± 3.18	24.91 ± 3.57	0.996a
Hypertension duration (years)	9.47 ± 11.92	6.14 ± 8.86	0.028a*
Hypertension, *n* (%)	49 (65.33)	61 (51.69)	0.188c
SBP (mmHg)	136.95 ± 20.85	137.47 ± 20.12	0.863a
DBP (mmHg)	80.89 ± 12.43	80.67 ± 11.73	0.900a
Diabetes duration (years)	11.91 ± 6.17	10.44 ± 5.75	0.093a
HbA1c (%)	9.46 ± 2.10	8.63 ± 2.06	0.008a*
FBG (mmol/L)	8.31 ± 2.73	8.05 ± 2.63	0.501a
2h-PBG (mmol/L)	15.00 ± 4.11	14.31 ± 4.22	0.260a
TG (mmol/L)	2.11 ± 2.16	1.70 ± 1.16	0.086a
TC (mmol/L)	4.82 ± 1.29	4.45 ± 1.05	0.031a*
LDL-c (mmol/L)	2.97 ± 0.94	2.75 ± 0.83	0.095a
HDL-c (mmol/L)	1.21 ± 0.33	1.13 ± 0.29	0.085a
Use of insulin (%)	41 (54.70)	71 (60.20)	0.450c
ApoA1 (g/L)	1.18 ± 0.28	1.10 ± 0.30	0.071a
ApoB (g/L)	0.88 ± 0.34	0.84 ± 0.42	0.513a
24-OHC (ng/L)	3.98 ± 1.25	3.56 ± 0.70	0.031b*
**Cognition test levels**			
MoCA	23 (21–24)	28 (27–29)	<0.001b*
DST	10.59 ± 1.84	12.30 ± 2.03	<0.001a*
VFT	14 (13–17)	17 (14–21)	<0.001b*
CDT	3.07 ± 0.92	3.52 ± 0.81	<0.001a*
TMT-A	73.15 ± 24.77	57.08 ± 20.38	<0.001a*
TMT-B	198 (152–240)	125.00 (101.50–160.50)	<0.001b*
SCWT A time	35.60 ± 12.91	31.19 ± 13.50	0.026a*
SCWT A number	49.63 ± 0.75	49.73 ± 2.31	0.711a
SCWT B time	58.57 ± 19.50	49.98 ± 21.41	0.002a*
SCWT B number	48 (46–50)	50 (49–50)	<0.001b*
SCWT C time	110.60 ± 33.74	90.27 ± 31.56	<0.001a*
SCWT C number	44.44 ± 4.37	47.14 ± 3.60	<0.001a*
AVLT IR	15.87 ± 4.46	19.53 ± 5.11	<0.001a*
AVLT DR	4.73 ± 2.20	6.50 ± 2.39	<0.001a*
LMT	6.85 ± 4.17	10.86 ± 4.26	<0.001a*
MMSE	27 (25–28)	29 (29–30)	<0.001b*

*p < 0.05.

*Data are presented as *n* (%), mean ± SD, or median (interquartile range) as appropriate.*

*a, Student’s *t*-test; b, Mann–Whitney *U* test; c, χ^2^ test; MCI, mild cognitive impairment; BMI, body mass index; SBP, systolic blood pressure; DBP, diastolic blood pressure; HbA1c, glycosylated hemoglobin; FBG, fasting blood glucose; 2h-PBG, 2-h post-meal blood glucose; TG, triglyceride; TC, total cholesterol; LDL-c, low-density lipoprotein cholesterol; HDL-c, high-density lipoprotein cholesterol; ApoA1, apolipoprotein A1; ApoB, apolipoprotein B; 24-OHC, 24S-hydroxycholesterol; MoCA, Montreal Cognitive Assessment; DST, digit span test; CDT, clock drawing test; VFT, verbal fluency test; TMT-A, trail making test-A; TMT-B, trail making test-B; SCWT, Stroop color and word test; AVLT_IR, auditory verbal learning test, immediately recall; AVLT_DR, auditory verbal learning test, delayed recall; LMT, logic memory test; MMSE, mini-mental state examination.*

### Partial Correlations of Plasma 24-OHC Concentrations With Neuropsychological Tests and TC

Given that neuropsychological test scores and plasma 24-OHC concentrations were obviously different, partial correlations of plasma 24-OHC concentrations with neuropsychological tests in patients with T2DM were analyzed (Table 2). Among the patients with diabetes, the plasma 24-OHC concentration was negatively correlated with the MoCA score after adjusting the age, gender, and educational attainment (*r* = −0.150, *p* = 0.039), further confirming that the plasma 24-OHC concentration could reflect a cognitive status. No other correlations were discovered in terms of MMSE, DST, VFT, TMTA, TMTB, SCWT A time, SCWT A number, SCWT B time, SCWT B number, SCWT C time, SCWT C number, AVLT IR, AVLT DR, and plasma 24-OHC concentrations between total patients and MCI patients. We also explored the relationship between 24-OHC and TC and found no correlation between them (*p* > 0.05).

**TABLE 2:** Partial correlations of plasma 24-OHC concentrations with neuropsychological tests and plasma level of TC in patients with T2DM.

Control variables		Total		MCI	
		
		*r*	*p*-Value	*r*	*p*-Value
MoCA	Age, gender, and educational attainment	–0.150	0.039*	0.033	0.785
MMSE		–0.085	0.241	0.048	0.692
DST		0.073	0.316	0.149	0.213
CDT		–0.079	0.276	–0.091	0.447
VFT		–0.066	0.364	–0.095	0.426
TMTA		–0.043	0.556	–0.098	0.412
TMTB		0.068	0.353	0.011	0.925
SCWT A time		–0.052	0.477	–0.120	0.316
SCWT A number		–0.011	0.876	0.098	0.415
SCWT B time		0.009	0.907	–0.04	0.739
SCWT B number		–0.076	0.299	–0.024	0.842
SCWT C time		0.099	0.174	0.041	0.735
SCWT C number		0.004	0.957	0.006	0.962
AVLT IR		0.037	0.611	0.074	0.539
AVLT DR		0.007	0.921	0.054	0.650
LMT		–0.057	0.437	–0.037	0.759
TC		0.2	0.782	0.014	0.904

*p < 0.05 (partial correlations were used here).

*T2DM, type 2 diabetes mellitus; MCI, mild cognitive impairment; TC, total cholesterol; MoCA, Montreal Cognitive Assessment; MMSE, mini-mental state examination; DST, digit span test; CDT, clock drawing test; VFT, verbal fluency test; TMT-A, trail making test-A; TMT-B, trail making test-B; AVLT_IR, auditory verbal learning test, immediately recall; AVLT_DR, auditory verbal learning test, delayed recall; LMT, logic memory test.*

### Distributions of the CYP46A1 Genotype and Allele Frequencies in the MCI and Control Groups

Table 3 shows the distributions of the CYP46A1 genotype and allele frequencies in the two groups. The distribution of the CYP46A1 genotype was consistent with the Hardy–Weinberg equilibrium in the MCI group and the control group. No difference was revealed in terms of distributions of the CYP46A1 genotype and allele frequencies between the MCI group and control group (*p* > 0.05).

**TABLE 3:** Distributions of the CYP46A1 genotype and allele frequencies between groups.

	Genotype, *n* (%)				Allele, *n* (%)		
		
Group	TT	TC	CC	*p*-Value	T	C	*p*-Value
MCI	37 (49.3)	29 (38.7)	9 (12)	0.985	103 (68.7)	47 (31.3)	0.858
Control	57 (48.3)	46 (39.0)	15 (12.7)		160 (67.8)	76 (32.2)	

*Data are presented as *n* (%). Chi-square test was used to compare the distribution of CYP46A1 genotype and allele frequencies between the two groups.*

*MCI, mild cognitive impairment.*

### Plasma Level of 24-OHC in Patients With T2DM

Table 1 shows a significant difference in plasma 24-OHC concentration between the MCI group and the normal cognitive group. Therefore, we explored whether the plasma 24-OHC concentration among patients with different genotypes in each group varies. As shown in Table 4, regardless of the MCI group, normal group, or total participants, the plasma 24-OHC concentration did not show a significant difference among the three genotypic subgroups. Patients with MCI and the CC genotype showed the highest plasma level of 24-OHC, followed by the TC and TT genotype, even though it was not statistically significant.

**TABLE 4:** Plasma level of 24-OHC (ng/L) in patients with T2DM, MCI, and normal cognition.

	Genotype			
	
Group	TT	TC	CC	*p*-Value
MCI	3.89 ± 0.86	3.92 ± 1.48	4.51 ± 1.78	0.390
Normal	3.59 ± 0.70	3.51 ± 0.61	3.61 ± 0.93	0.819
Total	3.71 ± 0.78	3.67 ± 1.05	3.95 ± 1.35	0.460

*24-OHC, 24S-hydroxycholesterol; T2DM, type 2 diabetes mellitus; MCI, mild cognitive impairment.*

*Data are presented as mean ± SD. One-way ANOVA was used to make a comparison of the plasma level of 24-OHC among different genotypes.*

### Comparison of Cognitive Performance Among Different Genotypes of the CYP46A1 Gene

To compare the cognitive differences among different genotypes, various neuropsychological test scores of the three genotypes (i.e., TT, TC, and CC) were compared, and the results are shown in Table 5. No significant differences were identified between genotypic subgroups in terms of neuropsychological test scores in the normal group (all *p* > 0.05). In the MCI group, VFT and SCWT A number were not the same in the three genotypic subgroups, and the difference was statistically significant (*p* = 0.019 and *p* = 0.033, respectively). Therefore, to further explore the specific differences between the two test scores in the three genotypic subgroups, LSD and all pairwise analyses were used (Table 6). The TT genotype had higher scores than the CC genotype, whether it was VFT or SCWT A number, suggesting that people with the CC genotype were more likely to have a cognitive impairment than those with the TT genotype (mean difference = 3.099 and 0.757; *p* = 0.008 and *p* = 0.029, respectively). Although the differences between TT and TC and between CC and TC were not statistically significant, people with more C genes had lower scores, suggesting that the C gene may be a risk factor of MCI.

**TABLE 5:** Comparison of cognitive performance according to genotypes of the CYP46A1 gene.

	MCI (*n* = 75)				Control (*n* = 118)			
		
	TT (*n* = 37)	TC (*n* = 29)	CC (*n* = 9)	*p*-Value	TT (*n* = 57)	TC (*n* = 46)	CC (*n* = 15)	*p*-Value
MoCA	23 (21–24)	23 (20–24)	23 (20.5–24)	0.559b	28 (27–28.5)	28 (27–29)	28 (26–29)	0.426a
LMT	6.86 ± 4.25	6.34 ± 4.37	8.44 ± 3.05	0.425a	10.95 ± 4.17	10.65 ± 4.12	11.20 ± 5.19	0.893a
AVLT IR	15.05 ± 3.47	16.86 ± 5.46	16.00 ± 4.39	0.122a	18.63 ± 4.93	19.87 ± 5.12	21.87 ± 5.22	0.077a
AVLT DR	4.32 ± 1.94	5.03 ± 2.34	5.44 ± 2.65	0.311a	6.05 ± 2.18	6.83 ± 2.52	7.20 ± 2.60	0.126a
VFT	15.43 ± 3.10	14.10 ± 2.94	12.33 ± 3.20	0.018a*	17.21 ± 4.25	18.30 ± 4.28	17.07 ± 4.10	0.375a
CDT	3.03 ± 1.01	3.24 ± 0.79	2.67 ± 0.87	0.248a	3.49 ± 0.85	3.61 ± 0.77	3.33 ± 0.82	0.499a
TMTA	71.03 ± 22.52	75.17 ± 26.14	75.33 ± 30.99	0.770a	58.12 ± 19.37	58.04 ± 23.15	50.13 ± 13.80	0.372a
TMTB	199.22 ± 87.74	210.79 ± 68.16	207.00 ± 91.39	0.844a	135.26 ± 39.86	143.57 ± 59.46	113.13 ± 43.92	0.116a
DST	10.54 ± 1.99	10.66 ± 1.76	10.56 ± 1.59	0.968a	12.46 ± 2.17	12.43 ± 1.88	11.27 ± 1.67	0.108a
MMSE	26.49 ± 2.10	25.93 ± 2.73	27.22 ± 1.30	0.309a	28.91 ± 0.95	29.22 ± 0.99	29.13 ± 0.83	0.261a
SCWT A time	32.05 ± 11.11	38.83 ± 14.65	39.78 ± 10.96	0.061a	31.09 ± 16.67	29.74 ± 8.14	36.07 ± 12.90	0.290a
SCWT A number	50 (50–50)	50 (50–50)	49 (48–50)	0.033b*	49.93 ± 0.26	49.41 ± 3.69	49.93 ± 0.26	0.498a
SCWT B time	56.68 ± 17.21	61.31 ± 22.00	57.56 ± 21.06	0.629a	47.49 ± 22.94	48.46 ± 18.33	56.27 ± 24.09	0.364a
SCWT B number	47.54 ± 2.61	47.86 ± 2.37	46.22 ± 2.86	0.247a	49.33 ± 1.17	48.78 ± 3.00	49.27 ± 1.10	0.393a
SCWT C time	106.51 ± 36.12	113.03 ± 33.62	119.56 ± 22.62	0.521a	90.58 ± 33.06	88.46 ± 29.51	94.57 ± 33.53	0.802a
SCWT C number	44.62 ± 4.96	44.48 ± 4.00	43.56 ± 3.00	0.809a	47.05 ± 3.99	47.07 ± 3.55	47.67 ± 1.92	0.832a

*p < 0.05.

*Data are presented as mean ± SD or median (interquartile range) as appropriate.*

*a, analysis of variance (ANOVA) was used to make a comparison of normally distributed quantitative variables between genotypic subgroups in MCI group and control group; b, Kruskal–Wallis test was used to make a comparison of asymmetrically distributed quantitative variables between genotypic subgroups in MCI group and control group.*

*MCI, mild cognitive impairment; MoCA, Montreal Cognitive Assessment; LMT, logic memory test; AVLT_IR, auditory verbal learning test, immediately recall; AVLT_DR, auditory verbal learning test, delayed recall, DST, digit span test; TMT-A, trail making test-A; TMT-B, trail making test-B; VFT, verbal fluency test; CDT, clock drawing test; MMSE, mini-mental state examination.*

**TABLE 6:** Pairwise comparison of neurological test scores in genotypic subgroups.

	VFT			SCWT A number		
		
	Mean difference	Standard error	*p*-Value^a^	Mean difference	Standard error	*p*-Value^b^
TT&CC	3.099	1.134	0.008*	0.757	0.268	0.029*
TT&TC	1.329	0.756	0.083	0.102	0.179	1.000
CC&TC	–1.770	1.164	0.133	–0.655	0.275	0.081

*P < 0.05.

*Data are presented as mean difference and standard error. Analysis of variance (ANOVA) for comparison of VFT and SCWT A number in genotypic subgroups.*

*^*a*^Using LSD for comparison of normally distributed quantitative variables. ^*b*^All pairwise for comparison of asymmetrically distributed quantitative variables.*

*VFT, verbal fluency test; SCWT, Stroop color and word test.*

### Logistic Regression Models

To find the risk factors related to MCI in patients with T2DM, we first conducted simple logistic regression and incorporated the independent variables including age, gender, education level, BMI, hypertension duration, SBP, DBP, HbA1c, diabetes duration, history of smoking and drinking, FBG, 2h-PBG, TG, TC, LDL-c, HDL-c, use of insulin, genotype, and 24-OHC. The results showed that the independent variables associated with the occurrence of MCI were age, gender, education level, hypertension duration, HbA1c, TC, and 24-OHC (Table 7).

**TABLE 7:** Assessment results of the risk of having MCI in a simple logistic regression model in patients with T2DM.

	β	SE	OR	*p*-Value	OR (95% CI)	
Age (years)	0.041	0.017	1.042	0.019*	1.007	1.078
Gender (female/male)	–1.001	0.305	0.367	0.001*	0.202	0.667
Education level (years)	–0.136	0.048	0.873	0.004*	0.795	0.958
BMI	0.000	0.043	1.000	0.996	0.918	1.088
Hypertension duration (years)	0.031	0.015	1.032	0.032*	1.003	1.062
SBP (mmHg)	–0.001	0.007	0.999	0.862	0.985	1.013
DBP (mmHg)	0.002	0.012	1.002	0.899	0.978	1.026
HbA1c (%)	0.189	0.073	1.208	0.010*	1.047	1.394
Diabetes duration (years)	0.042	0.025	1.043	0.095	0.993	1.095
Smoking, *n* (%)	–0.419	0.324	0.658	0.197	0.349	1.242
Drinking, *n* (%)	–0.345	0.387	0.709	0.374	0.332	1.514
Use of insulin, *n* (%)	–0.225	0.299	0.798	0.450	0.445	1.433
FBG (mmol/L)	0.037	0.055	1.038	0.499	0.932	1.157
2h-PBG (mmol/L)	0.040	0.035	1.041	0.259	0.971	1.116
TG (mmol/L)	0.170	0.108	1.186	0.115	0.959	1.465
TC (mmol/L)	0.283	0.135	1.327	0.036*	1.019	1.728
HDL-c (mmol/L)	0.836	0.489	2.307	0.088	0.884	6.020
LDL-c (mmol/L)	0.284	0.171	1.329	0.096	0.951	1.858
TT				0.985		
TC	–0.029	0.317	0.971	0.927	0.521	1.809
CC	–0.079	0.472	0.924	0.867	0.367	2.329
24-OHC (ng/L)	0.478	0.177	1.613	0.007*	1.140	2.282

*p < 0.05 (simple logistic regression was used).

*MCI, mild cognitive impairment; T2DM, type 2 diabetes mellitus; BMI, body mass index; SBP, systolic blood pressure; DBP, diastolic blood pressure; HbA1c, glycosylated hemoglobin; FBG, fasting blood glucose; 2h-PBG, 2-h post-meal blood glucose; TG, triglyceride; TC, total cholesterol; LDL-c, low-density lipoprotein cholesterol; HDL-c, high-density lipoprotein cholesterol; 24-OHC, 24S-hydroxycholesterol.*

Multivariable logistic regression was used, which showed that gender, education level, hypertension duration, HbA1c, and 24-OHC played a key role in the occurrence of MCI in patients with T2DM (Table 8).

**TABLE 8:** Assessment results of the risk of having MCI in a multivariable logistic regression model in patients with T2DM.

	β	SE	OR	*p*-Value	OR (95% CI)	
Gender (female/male)	–1.083	0.340	0.339	0.001*	0.174	0.659
Education level (years)	–0.134	0.053	0.874	0.011*	0.788	0.970
Hypertension duration (years)	0.049	0.017	1.050	0.003*	1.017	1.085
HbA1c (%)	0.249	0.081	1.283	0.002*	1.094	1.504
24-OHC (ng/L)	0.614	0.193	1.848	0.001*	1.266	2.697

*p < 0.05 (multivariable logistic regression was used).

*MCI, mild cognitive impairment; T2DM, type 2 diabetes mellitus; HbA1c, glycosylated hemoglobin; 24-OHC, 24S-hydroxycholesterol.*

## Discussion

Our study demonstrated that plasma 24-OHC concentration played a negative role in the development of T2DM-associated MCI, as high levels were observed in patients with T2DM and MCI but low levels were noted in patients with T2DM and normal cognition. This result was further confirmed by our analysis, which revealed a negative correlation between the plasma 24-OHC concentration and MoCA after adjusting for age, gender, and educational attainment. No association between the genotype of CYP46A1 rs754203 and the plasma level of 24-OHC was found. Although the *p*-value was more than 0.05, patients with MCI and the CC genotype had the highest plasma level of 24-OHC among the tested patients. Among the patients with diabetes, those who carry a CC genotype may have a greater risk of developing MCI when compared to patients with a TT genotype. Logistic regression showed that females, low education level, long hypertension duration, and high HbA1c and plasma 24-OHC concentrations were associated with MCI in patients with T2DM. There was neither a significant difference in distributions of the CYP46A1 genotype between the MCI group and control group nor in plasma 24-OHC concentration among the three genotype subgroups.

Previous studies reported elevated plasma 24-OHC concentration levels in patients with MCI and AD (Lütjohann et al., 2000; Zuliani et al., 2011), which was consistent with our results. This conclusion supports that the plasma level of the 24-OHC concentration is proportional to the degree of brain atrophy and the loss of active gray matter (Liu et al., 2016). However, other studies indicated that people with AD have low levels of plasma 24-OHC concentration (Bretillon et al., 2000; Solomon et al., 2009; Burlot et al., 2015), and no significant difference was found between the MCI group and normal group (Liu et al., 2016). The possible explanation for this contradiction may be that the patients of these studies were at different stages of the disease. One previous research reported that the highest plasma level of 24-OHC was observed in mild AD patients (Li et al., 2018). When brain tissue damage is less severe in the early stage of the disease (such as MCI and early AD), the 24-OHC in plasma obviously rises but declines at the advanced stage of the disease. This phenomenon may be due to the decreased cholesterol turnover in the brain in the late stages, after a large number of neurons have degenerated and died, resulting in a low production of 24-OHC (Papassotiropoulos et al., 2000; Testa et al., 2016).

Besides 24-OHC, 27-hydroxycholesterol (27-OHC) is also an important oxysterol in the human body. It is a selective estrogen receptor modulator, which may affect tissues containing estrogen receptors such as blood vessels, bones, and breasts (Umetani and Shaul, 2011). It is derived from the periphery and can reflect the oxidative metabolism of cholesterol in the periphery (Hughes et al., 2013). Given that it can enter the brain through the blood–brain barrier, 27-OHC has also been shown to be neurotoxic and influences the occurrence of neurological disease (Popp et al., 2012; Chen et al., 2019). However, its effects within the brain remain unclear, and it is not expected to contribute to the pool of cholesterol in the brain (Hughes et al., 2013). Other studies have reported that it is also related to many non-neurological diseases, such as breast cancer, prostate cancer, lung adenocarcinoma, and COVID-19 (Marwarha et al., 2017; Zhang et al., 2019; Marcello et al., 2020). Therefore, compared with 24-OHC, 27-OHC seems to lack a certain specificity for cognitive disorders. That is why we chose 24-OHC as a priority for exploration. However, the influence of 27-OHC in neurological diseases cannot be ignored, and further research on it in the future is necessary.

In previous studies, the relationship between the CYP46A1 rs754203 polymorphism and MCI or AD has been controversial. Some studies have reported that the TT genotype and T allele are risk factors for the incidence of AD (He et al., 2012), while other studies suggested the CC genotype and C allele instead (Golanska et al., 2005). Others reported no correlation between them (Desai et al., 2002). Our results showed no difference in the distribution of the three genotypes between the MCI and the control groups. Although we obtained negative results in genotype distribution, when exploring the relationship between the polymorphism of the CYP46A1 rs754203 gene and the scores of various cognitive tests, VFT and SCWT A number showed significant differences among the three genotype subgroups of the MCI group. VFT is commonly used to assess lexical access from orthographic and phonemic networks, which is useful to measure executive function (Henry et al., 2004). The SCWT is a neuropsychological test extensively used for both experimental and clinical purposes (Scarpina and Tagini, 2017). SCWT A TEST is a word task, which can measure selective attention, cognitive flexibility information, and cognitive inhibition (Rivera et al., 2017). Pair comparison revealed that the scores of patients with the CC genotype were significantly lower than those with the TT genotype. Interestingly, when pair comparisons of TT and TC and of CC and TC were conducted, patients with the C allele had lower scores on these two tests despite the *p*-value being greater than 0.5. Therefore, we speculate that the CC genotype of CYP46A1 rs754203 might be a risk factor for diabetic MCI. The reason why there was no difference in the distribution of the three genotypes in our study may be related to the small number of participants. Our patients were all from the same hospital in China, making the study relatively limited in regions. The distribution of gender and age between the two groups was uneven, which possibly affected the results of the study to some extent. Li et al. (2013) pointed out that the presence of APOE ε4 allele can strengthen the effect of the CC genotype on AD risk. However, this gene was not detected in our study due to technical problems, and this factor was not considered when analyzing the results, which may have also affected our results. This should be taken into account in our subsequent studies.

No significant difference in plasma 24-OHC was found among the three genotype subgroups in our research. Although the *p*-value was greater than 0.05, we observed that the carriers of the CC genotype in the MCI group had the highest plasma 24-OHC, followed by the TC genotype and TT genotype; these findings were completely opposite to a previous study. In a previous study, what confused the researchers was that they found that people with the CC genotype had a higher risk of developing AD, but those with the TT genotype seemed to show a higher plasma concentration of 24-OHC (Li et al., 2018). An accurate explanation was not explored, and they speculated that this result may mean that other mechanisms may exist between the CYP46A1 gene polymorphism and the pathology of diseases related to AD and MCI.

A close relationship exists among AD, MCI, and T2DM. Janson et al. (2004) suggested a strong link between the neurodegenerative processes that lead to the loss of cortical brain cells in AD and the loss of β-cells in T2DM. Many previous studies, including our study, have explored the relationship between CYP46A1 gene polymorphisms and AD or MCI and reached some inconsistent conclusions. Some studies focused on the relationship between the CYP46A1 gene polymorphism and T2DM. In Rizvi et al.’s (2017) study, CC, CT genotypes, and C allele were found to be positive risk factors for T2DM. On the basis of these interesting findings, we suspect that patients with the CC genotype are more likely to develop T2DM, so people with the CC genotype among those with T2DM are more severely ill and lose more islet cells; thus, they are more likely to develop MCI. Lipid oxidation may be one of the body’s responses to islet dysfunction. This may explain some of the possible mechanisms between the CYP46A1 gene polymorphism and the occurrence of cognitive impairment.

According to the data in Table 1, the patients with MCI were older, with more female cases than males, having a shorter time of education, with higher HbA1c and 24-OHC levels, and suffering from hypertension longer than those with normal cognition. These findings showed that these factors may be related to the onset of MCI. Subsequent logistic regression analysis, after controlling for confounding factors, further proved this relationship and found these factors to be independent risk factors of MCI in patients with T2DM. For these factors, related studies have been conducted in the past. For age, some found that the incidence of MCI increases with age (Roberts et al., 2012; Ward et al., 2012), while others have not found the overall age influence (Brodaty et al., 2013). Regarding the impact of gender, controversies persist. Some believe that there is no significant difference in incidence between genders (Overton et al., 2019), and some studies believe that women are at a higher risk than men (Ma et al., 2016). For hypertension, previous studies showed that long-term high blood pressure status can cause brain material atrophy or white matter damage, which is closely related to cognitive impairment (Nagai et al., 2008, 2010). In terms of education, numerous studies believe that a high level of education can reduce the risk of developing AD; in the MCI stage, education may have a protective effect on total brain volume (Sattler et al., 2012), thereby preventing the occurrence of cognitive impairment (Wada et al., 2018). The US National Institutes of Health highlighted diabetes mellitus, smoking, depression, mental inactivity, physical inactivity, poor diet, hypertension, obesity, and low educational attainment as risk factors of cognitive decline and AD (Daviglus et al., 2010; Barnes and Yaffe, 2011). Our findings are consistent with previously reported results; some of the factors were not covered in our research, so they can be further confirmed in future work.

Similar studies were mostly based on people with no other particular diseases, whereas our study was conducted in patients with T2DM, which is a different point of innovation in our research. Our findings provide experimental evidence to some extent to reveal the pathogenesis of diabetes-related MCI, include some reference for future related research, and identify the possible indicators for early diagnosis and discovery of T2DM-associated MCI. Some possible risk factors for MCI were determined to help better prevent type 2 diabetic MCI. Despite these achievements, our research still had some shortcomings. First, many methods can be used for the determination of oxysterol, including gas chromatography–mass spectrometry, liquid chromatography–mass spectrometry, and liquid chromatography electro-spray ionization tandem mass spectrometry (Griffiths et al., 2013; Karuna et al., 2015). However, as a result of the limitations of our experimental technology, we could not determine the concentration of 24-OHC by these methods. We finally chose the ELISA method because its sensitivity and accuracy are slightly inferior to other methods. The literature revealed other studies that used the ELISA method to determine the plasma 24-OHC concentration (Roy et al., 2019). We used double-antibody sandwich ELISA, which has high sensitivity and high specificity, to maximize the reliability of the measurement results. Second, this work is a cross-sectional study, and the prevalence of MCI in the overall population was only 21.4% (Overton et al., 2019). The sample size of the participants and the number of patients with MCI and diabetes were both small, leading to poor matching of the two groups in terms of some baseline characteristics, such as age, education level, and hypertension duration. This possibly affected our results to a certain extent. Thus, when performing correlation analysis and logistic regression analysis, we adopted relevant statistical methods to control these confounding factors to minimize their possible impact. Third, our research was only carried out in patients with T2DM, and a group of non-diabetic people was lacking. Related research in these people must be carried out in the future to make the results more comprehensive. Fourth, the APOE ε4 allele is a key factor in the onset of AD and MCI; it exerts a remarkable influence on the relationship between the polymorphism of CYP46A1 and the onset of cognitive impairment (Li et al., 2013). However, our study did not detect this gene, so our research results lack accuracy and comprehensiveness. Lastly, our results showed no difference in the distribution of TT, CC, and TC between the two study groups. Only two cognitive-related test scores were different among the three subgroups, so we concluded that the CC genotype could lead to a higher incidence of MCI than the other genotypes. However, this result needs further scrutiny and should be interpreted with caution. Moreover, we only extracted the peripheral blood instead of the cerebrospinal fluid for testing due to operational inconvenience, so we can only speculate the situation of central lipid metabolism based on the results measured from the periphery.

## Conclusion

In conclusion, early diagnosis of MCI is crucial to prevent further progression to AD. Our research have found that, in patients with T2DM, high plasma level of 24-OHC and the CC genotype carrier of CYP46A1 rs754203 may portend a high risk of developing early cognitive impairment, including attention and executive deficits. However, research based on a large sample size and wide population should be further conducted to explore the potential mechanisms.

## Data Availability Statement

All data generated or analyzed during this study are included in this article.

## Ethics Statement

The studies involving human participants were reviewed and approved by the Research Ethics Committee of the Affiliated Zhongda Hospital of Southeast University. The patients/participants provided their written informed consent to participate in this study.

## Author Contributions

SW and YY contributed to the idea and revised the manuscript. JS carried out the design, conducted the study, and wrote the manuscript. WC and KA carried out the data collection. JJ and ST participated in the data analysis. HZ and WZ helped with data interpretation. All the authors read and approved the final manuscript.

## Conflict of Interest

The authors declare that the research was conducted in the absence of any commercial or financial relationships that could be construed as a potential conflict of interest.
